# IV-EVE: EVE for Injection—ADME, Pharmacokinetic and Toxicological Evaluation for Novel Deciparticle EVE Formulation

**DOI:** 10.3390/biomedicines14071573

**Published:** 2026-07-14

**Authors:** Wen-Han Chang, Nancy Chang, Sheng-Hao Min, John M. Lopp, Robert Hoff, Tanjina Hoque, Vuong Trieu, Cynthia Lee

**Affiliations:** 1 Sapu Bioscience LLC, 10840 Thornmint Road, Suite 118, San Diego, CA 92127, USA; wenhan.chang@sapubio.com (W.-H.C.); nancy.chang@sapubio.com (N.C.); jack.min@sapubio.com (S.-H.M.); john.lopp@sapubio.com (J.M.L.); robert.hoff@sapubio.com (R.H.); tanjina.hoque@sapubio.com (T.H.); 2 Oncotelic Therapeutics Inc., 29397 Agoura Road, Suite 107, Agoura Hills, CA 91301, USA; vtrieu@oncotelic.com; 3 Sapu Nano (US) LLC, 10801 Thornmint Rd. Suite 150, San Diego, CA 92127, USA

**Keywords:** EVE, pharmacokinetics, toxicology, mTOR, cancer therapy

## Abstract

**Background**: Oral everolimus (Oral-EVE; EVE, Afinitor^®^) is an effective therapy for multiple malignancies, including HR-positive/HER2-negative breast cancer, but its clinical use is limited by low and variable oral bioavailability as well as extensive first-pass metabolism. IV-EVE is a Deciparticle™ intravenous formulation of everolimus developed to bypass gastrointestinal absorption and improve systemic pharmacokinetic (PK) predictability. **Methods**: In vitro ADME properties were assessed across multiple species. IV-EVE (Intravenous EVE, Sapu003) and EVE demonstrated comparable plasma protein binding, metabolic stability, and CYP-mediated metabolism. Pharmacokinetics, tissue distribution, metabolism, and excretion of IV-EVE were evaluated following intravenous administration in rats, with Oral-EVE as a comparator. **Results**: Elimination of intact everolimus was minimal following either route of administration and occurred predominantly through metabolic biliary/fecal pathways. Compared with Oral-EVE, IV-EVE produced substantially higher and more consistent systemic exposure while maintaining comparable elimination half-lives. Intravenous administration resulted in broad tissue distribution without the marked gastrointestinal accumulation observed following oral dosing. Repeat-dose toxicology studies in rats demonstrated no treatment-related gastric pathology following intravenous administration. **Conclusions**: These findings suggest that intravenous administration may overcome limitations associated with oral delivery and support the clinical evaluation of IV-EVE in patients with advanced malignancies.

## 1. Introduction

EVE (RAD001, Afinitor; 40-O-(2-hydroxyethyl)-rapamycin) is a synthetic, orally available analogue of rapamycin (sirolimus) [1,2]. Functionally, it is a potent, oral inhibitor of the mammalian target of rapamycin (mTOR) that binds FKBP12 and inhibits the mTORC1 complex and therefore is not expected to induce rapid cell death but rather to slow tumor growth [2]. EVE is characterized by inhibition of mTORC2 at high concentrations, where rapamycin is completely devoid of mTORC2 activity. EVE has been investigated in multiple phase 3 clinical trials since 1996 after being initially developed for the prophylaxis of organ transplant rejection [2]. Pre-clinically, EVE inhibits the proliferation of various human tumor cell lines in vitro and diminishes HIF1/VEGF expression, activities that translate into confirmed in vivo anti-angiogenic effects [2].

EVE is now an approved oncology agent [3] and the first oral inhibitor of mTOR to reach the oncology clinic [4]. Regulatory approvals now span a broad spectrum of malignancies and rare diseases. In the European Union, the EMA has authorized Afinitor for hormone-receptor-positive, HER2-negative advanced breast cancer (with exemestane); progressive pancreatic, gastrointestinal or lung neuroendocrine tumors; and advanced renal cell carcinoma; Votubia is approved for renal angiomyolipoma and subependymal giant-cell astrocytoma (SEGA) associated with tuberous sclerosis complex (TSC) [5]. U.S. FDA approvals include advanced breast cancer, progressive pancreatic neuroendocrine tumors, advanced renal cell carcinoma after failure of sunitinib or sorafenib, renal angiomyolipoma/TSC, and SEGA/TSC [5].

Several class-specific effects, primarily metabolic and pulmonary toxicities, have occurred with rapamycin and its analogues [6]. These include hyperglycemia: The ARCC trial reported hyperglycemia in 26% of patients. In RECORD-1, hyperglycemia in 57% and grade 3 or 4 glucose intolerance in 15% was reported [7]. Hyperlipidemia: With temsirolimus, hypercholesterolemia and hypertriglyceridemia in 21% and 25%, with primarily grades 1–2 [8]. With EVE, cholesterol and triglyceride levels were elevated in 77% and 73% (the majority grades 1–2) [7]. Hypophosphatemia: Mild hypophosphatemia has been reported in 37% for EVE in phase III trials [7,8]. Noninfectious pneumonitis: For EVE (RECORD-1), incidence was13.5% (3.6% grade 3, none grade 4) with a median time to occurrence of 108 days [7].

In a multi-center RCC cohort, stomatitis (56 vs. 30%, *p* < 0.001) and non-infectious pneumonitis (38 vs. 22%, *p* = 0.018) were more frequently observed with EVE than temsirolimus (IV-Sirolimus), while asthenia (11 vs. 23%, *p* = 0.027), rash (20 vs. 36%, *p* = 0.018), and fatigue (33 vs. 48%, *p* = 0.032) occurred less frequently with EVE [9]. Hyperlipidemia and hyperglycemia were numerically higher with EVE (77%/73% lipids; 57% glucose) than with temsirolimus (21%/25% lipids; 26% hyperglycemia) in their pivotal trials [7,8]. Hypophosphatemia was recorded as follows: 37% EVE vs. 6% temsirolimus [7,8]. Hematologic toxicity profiles were comparable: EVE (all-grade)—anemia 61.2%, thrombocytopenia 36.0%, lymphopenia 40.9%, and neutropenia 21.7% [10]; temsirolimus (phase III RCC; all-grade)—anemia 93.8%, thrombocytopenia 40.4%, lymphopenia 52.9%, and neutropenia 18.8% [10].

EVE is constrained by frequent and sometimes severe GI and oral mucosal toxicities—particularly stomatitis—which are dose-related, often early, and significantly drive dose modifications and discontinuation [11,12,13]. Once-daily regimens in oncology are often associated with treatment-limiting toxicity, motivating evaluation of divided-dose strategies [14]. Additional limitations include variable oral bioavailability and pharmacokinetic variability [15]. These limitations emphasize the need for close clinical monitoring and highlight the importance of alternative drug delivery strategies aimed at minimizing GI and mucosal toxicities while preserving therapeutic efficacy. In the present study, we report the development of a novel IV formulation of EVE (IV-EVE) designed to overcome the limitations of oral administration.

## 2. Materials and Methods

### 2.1. Test Article and Formulation

IV-EVE (Sapu003) was supplied by Sapu Bioscience, LLC (San Diego, CA, USA), and everolimus powder was purchased from Selleckchem (Houston, TX, USA). The test articles were stored at 2–8 °C and protected from light until use. IV-EVE is manufactured as sterile-filtered, lyophilized intravenous everolimus Deciparticle™ micelles using an mPEG-Chol system as previously described [16]. IV-EVE is a sterile, reproducible, nanoscale intravenous everolimus formulation with controlled particle size, defined drug loading, acceptable residual solvent, appropriate pH and water content, and stability characteristics suitable for clinical evaluation. Across cGMP clinical batches, reconstituted particle size was found to be 13.1–13.9 nm, and all tested batches met the predefined particle size specification of <30 nm. At the selected 1:5 drug-to-polymer ratio, the calculated everolimus mass fraction is approximately 16.7%. No visible everolimus precipitation was observed, and drug loading was therefore treated as effectively complete under the tested conditions. Release testing of cGMP batches included everolimus assay, residual ethanol, particle size, pH, and water content. Initial assay values ranged from 94.5% to 98.4%, pH ranged from 5.0 to 5.2, water content ranged from 1.4% to 1.6%, residual ethanol ranged from 75.6 to 115.7 ppm, and particle sizes remained within specification. In-use stability data showed that the reconstituted dosing solution remained <20 nm under clinically relevant handling conditions. Long-term stability supported refrigerated storage at 2–8 °C, whereas elevated temperature and humidity accelerated everolimus degradation. Sterility and endotoxin testing confirmed a sterile drug product that is endotoxin-free.

### 2.2. IV-EVE (Sapu003) Clinical Production

Everolimus (API) (BrightGene Pharma, Suzhou, China) was used as the active pharmaceutical ingredient for preparation of the Sapu003 nanoparticle formulation. The amphiphilic carrier polymer mPEG-Chol was used as the nanoparticle-forming excipient. Absolute ethanol (200 proof) (Fisher Scientific, Waltham, MA, USA) served as the organic solvent for drug–polymer dissolution. Anhydrous lactose (Spectrum Chemical, New Brunswick, NJ, USA) was used as a stabilizing excipient. Water for Injection (WFI) (Cytiva, Marlborough, MA, USA) was used for preparation of the aqueous phase. All reagents were of a pharmaceutical or analytical grade and used as received. All processing was performed under controlled lighting conditions to minimize photodegradation of everolimus.

Sapu003/everolimus for injection bulk nanoparticle formulation was manufactured under a GMP environment. The target formulation strength of the bulk dispersion was 4.0 mg/mL everolimus, corresponding to a 12 g drug load per batch. The manufacturing process consisted of polymer melting, preparation of a drug–polymer solution in ethanol, formation of nanoparticles through controlled aqueous dispersion, bulk homogenization, sterile filtration, and refrigerated storage of the final bulk formulation.

Everolimus was added to the polymer–ethanol solution under amber light conditions to minimize potential photodegradation. The mixture was vortex-mixed and subsequently agitated on an orbital shaker until the drug was completely dissolved and a homogeneous solution was obtained. The resulting drug–polymer solution was transferred to a sterile reaction flask for nanoparticle formation.

An aqueous lactose stabilizing solution was prepared in a separate sterile vessel. Water for Injection was combined with anhydrous lactose. The solution was stirred on a magnetic stir plate for approximately 10 min to reach homogeneity. Nanoparticle formation was initiated by slowly adding the aqueous lactose solution to the drug–polymer ethanol solution under continuous mixing. The mixture was rotated and mixed during which nanoparticle formation occurred and the dispersion became visually clear. This process produced a homogeneous bulk nanoparticle dispersion containing everolimus.

The bulk nanoparticle dispersion was subjected to sterile filtration using non-pyrogenic sterile vacuum filtration units. Process achieved recovery of approximately 96–100% relative to the theoretical batch mass. Following sterile filtration, the bulk formulation was aseptically filled into depyrogenated vials and lyophilized to produce the final drug product. Lactose served as a lyo-protectant and bulking agent to preserve nanoparticle integrity during freezing and drying, improve cake structure, and facilitate rapid reconstitution. The final clinical presentation targeted 20 mg everolimus per vial. Prior to administration, the lyophilized product was reconstituted with Water for Injection to yield a final everolimus concentration of approximately 4 mg/mL.

Sapu003 drug products were tested through multiple release tests, including identity and potency, as well as mean particle diameter and polydispersity index. Everolimus identity and content were quantified using an Agilent 1260 Infinity II HPLC (Agilent Technologies, Santa Clara, CA, USA) with quaternary pump, autosampler, thermostatic column compartment, and VWD. An Agilent Zorbax Eclipse XDB-C18 (Agilent Technologies, Santa Clara, CA, USA), 3 mm × 250 mm reverse phase column was used, with aqueous 0.27 g L^−1^ potassium monophosphate and 100% methanol chosen as the weak and strong mobile phases respectively. Samples were diluted to a theoretical everolimus concentration of 20 µg mL^−1^ in 40% aqueous acetonitrile and stored in amber 2 mL HPLC vials at 4 °C for no more than 48 h. The absorbance of everolimus and its Oxepane isomer was measured at 275 nm at a flow rate of 1.1 mL min^−1^ and a gradient method. Potency was determined by a three-point calibration using a working standard provided by a primary US Pharmacopeia (USP) standard or a secondary standard of API qualified against USP reference material. The Z-average particle diameter and polydispersity index of the micelle nanoparticles were determined by dynamic light scattering (Malvern Zetasizer, ZEN3600, Malvern, Worcestershire, UK) at an angle of 173° at room temperature. Drug products were reconstituted in ultrapure water and diluted 10-fold before analysis.

The stability of Sapu003 formulations was evaluated under both storage and in-use conditions using particle size (Z-average) and polydispersity index (PDI) as critical quality attributes. Chemical stability was also evaluated via HPLC, pH measurement and water content testing. For storage stability, lyophilized or formulated samples were stored under the following conditions: 2–8 °C, 25 °C/60% RH ± 5% RH, and 30 °C/65% RH ± 5% RH. At each condition, samples were taken from the stability chambers and subjected to predefined tests. Everolimus assay percent was tested using Agilent 1260 Infinity II HPLC; water content percent was tested via Karl Fischer Titrator. For solution acidity and particle size distribution, samples were reconstituted at predefined time points and analyzed by pH meter and dynamic light scattering (DLS) respectively. For in-use stability, formulations were first reconstituted in Water for Injection (WFI) and evaluated immediately (0 h), followed by storage under the same three temperature conditions (2–8 °C, 25 °C/60% RH ± 5% RH, and 30 °C/65% RH ± 5% RH). Particle size and PDI were measured at 0 h, 24 h, 7 days, and 14 days post-reconstitution. Stability was assessed based on the ability of the formulation to maintain particle size within the predefined acceptance criteria (<20 nm) and PDI < 0.2, indicating preservation of micelle integrity and formulation homogeneity over time under the tested conditions.

### 2.3. In Vitro Plasma Stability

The in vitro plasma stability of IV-EVE was evaluated in pooled plasma obtained from CD-1 mice, Sprague-Dawley rats, beagle dogs, cynomolgus monkeys, and human donors. Plasma samples were collected into K_2_-EDTA anticoagulant tubes and maintained under appropriate storage conditions until use. Plasma stability studies were conducted to compare degradation kinetics of the formulated intravenous product with those of unformulated everolimus under physiologically relevant conditions. IV-EVE was evaluated as the final clinical formulation. The lyophilized drug product (20 mg/vial) was reconstituted with sterile saline to generate a 1.0 mM stock solution. Conventional everolimus was evaluated using reference-grade drug substance (99.77% purity), which was dissolved in dimethyl sulfoxide (DMSO) to prepare a 10.0 mM stock solution. Working solutions were subsequently prepared at 0.100 mM. For IV-EVE, the working solution was generated by diluting the saline stock solution with saline, whereas conventional everolimus working solutions were prepared by dilution in DMSO. Thus, IV-EVE was tested as the finished intravenous formulation, while conventional everolimus was evaluated as the unformulated active pharmaceutical ingredient. Prior to incubation, plasma samples were equilibrated at 37 °C for 5 min. Reactions were initiated by adding 1 μL of 0.100 mM test article working solution to 99 μL of pre-warmed plasma, resulting in a nominal final everolimus concentration of approximately 1 μM. Samples were incubated at 37 °C and collected at 0, 5, 15, 30, 60, and 120 min. All experimental conditions were performed in triplicate. For time-zero controls, quenching solution was added before compound addition to prevent incubation-dependent degradation. For all remaining time points, reactions were terminated at the designated sampling intervals by addition of ice-cold protein precipitation solution. Reaction quenching was achieved using acetonitrile containing tolbutamide (100 ng/mL) and buspirone hydrochloride (10 ng/mL) as internal standards. Following protein precipitation, samples were mixed at 600 rpm for 5 min and centrifuged at 6000 rpm for 15 min at 4 °C. Aliquots of the resulting supernatant were diluted 1:1 with ultrapure water prior to bioanalysis. Everolimus concentrations were quantified by validated LC-MS/MS methods. Analyte responses were expressed as the peak area ratio of everolimus to internal standard. The percentage of parent compound remaining at each time point was calculated relative to the corresponding time-zero sample. Log-transformed percent remaining values were fitted by linear regression to determine the first-order elimination rate constant (k), and apparent plasma half-life (t½) was calculated according to: t½ = 0.693/k. Compounds demonstrating negligible decline over the 120 min incubation period were considered stable and assigned a half-life greater than 120 min. To verify assay performance, species-specific positive control substrates with known plasma instability were included. Procaine was used for mouse, monkey, and human plasma; benfluorex for rat plasma; and bisacodyl for dog plasma. Rapid degradation of the positive controls confirmed the metabolic competence of each plasma system and the suitability of the assay for evaluating everolimus stability.

### 2.4. In Vitro Metabolic Stabilities in Liver Microsomes and Hepatocytes

The metabolic stability of IV-EVE was evaluated in liver microsomes and cryopreserved hepatocytes obtained from CD-1 mice, Sprague-Dawley rats, beagle dogs, cynomolgus monkeys, and human sources. Metabolic turnover was assessed using validated LC-MS/MS methods to compare the intrinsic hepatic metabolism of the finished intravenous formulation with that of the unformulated everolimus drug substance. IV-EVE was evaluated as the final drug product formulation. Lyophilized drug product (20 mg/vial) was reconstituted with sterile saline to generate a 1.0 mM stock solution. Conventional everolimus was evaluated as a reference-grade active pharmaceutical ingredient (99.77% purity) dissolved in dimethyl sulfoxide (DMSO) to generate a 10.0 mM stock solution. Accordingly, the intravenous formulation was tested as the finished clinical product following reconstitution, whereas conventional everolimus was tested as an unformulated drug substance. Liver microsomes were supplied at a protein concentration of 20 mg/mL and diluted to a final incubation protein concentration of 0.5 mg/mL. For each species, IV-EVE and conventional everolimus were diluted to a final incubation concentration of 1.0 μM. Test compounds were first prepared as 500 μM working solutions and subsequently mixed with liver microsomes in phosphate-buffered saline (PBS). Incubation mixtures were pre-equilibrated at 37 °C prior to reaction initiation. Metabolism was initiated by addition of NADPH tetrasodium salt to achieve a final NADPH concentration of 2.0 mM. Negative control incubations lacking NADPH were performed in parallel to assess non-enzymatic degradation. No additional cofactors or regenerating systems were added. Microsomal incubations were conducted in 45 μL total reaction volume containing 1.0 μM test article, 0.5 mg/mL microsomal protein, and 2.0 mM NADPH. Samples were incubated at 37 °C and collected at 0, 5, 15, 30, 45, and 60 min. Reactions were terminated by addition of acetonitrile containing tolbutamide and buspirone internal standards. Following protein precipitation, samples were centrifuged and the resulting supernatants analyzed by LC-MS/MS. Verapamil was used as the positive control substrate in microsomal assays. Hepatocytes were washed, resuspended in Williams’ Medium E, and assessed for viability by trypan blue exclusion. Cells were diluted to a final viable cell density of 0.5 × 10^6^ cells/mL and pre-equilibrated at 37 °C before compound addition. IV-EVE and conventional everolimus were tested at a final concentration of 1.0 μM. Test compounds were prepared as 100 μM working solutions and added to hepatocyte suspensions to initiate metabolism. Incubations were performed in Williams’ Medium E under physiological culture conditions at 37 °C with 5% CO_2_, greater than 90% relative humidity, and orbital shaking at 350 rpm. Vehicle control incubations lacking hepatocytes were included to assess non-cellular degradation of the test articles. The final incubation system consisted of 1.0 μM test article and 0.5 × 10^6^ viable hepatocytes/mL. Samples were collected at 0, 15, 30, 60, 90, and 120 min and immediately quenched with acetonitrile containing internal standards. Following centrifugation, supernatants were diluted with ultrapure water and analyzed by LC-MS/MS. Testosterone and 7-hydroxycoumarin served as positive controls in hepatocyte studies. Parent compound depletion was quantified using validated LC-MS/MS assays. Everolimus concentrations were expressed as analyte-to-internal standard peak area ratios, and the percentage of parent compound remaining at each time point was calculated relative to time zero. Log-linear regression analysis was used to determine first-order elimination rate constants and metabolic half-lives. In vitro intrinsic clearance, predicted in vivo intrinsic clearance, hepatic clearance, and hepatic extraction ratios were subsequently estimated using standard well-stirred liver models.

### 2.5. CYP450 Phenotyping Studies

The cytochrome P450 (CYP) enzymes were investigated using complementary recombinant enzyme and chemical inhibition approaches in accordance with contemporary regulatory guidance for in vitro drug interaction studies. IV-EVE was evaluated as the final clinical formulation. Lyophilized drug product (20 mg/vial) was reconstituted in saline to generate a 1.0 mM stock solution. Conventional everolimus was evaluated as an unformulated active pharmaceutical ingredient (99.77% purity) dissolved in dimethyl sulfoxide (DMSO) to generate a 10.0 mM stock solution. Both test articles were diluted to a final incubation concentration of 1.0 μM for all phenotyping experiments. For recombinant CYP enzyme phenotyping, recombinant CYP1A2, CYP2B6, CYP2C8, CYP2C9, CYP2C19, CYP2D6, and CYP3A4 preparations were incubated individually with either IV-EVE or conventional everolimus. Incubations were conducted in phosphate-buffered saline (PBS) in the presence of NADPH as the metabolic cofactor. Following a 5 min pre-equilibration period at 37 °C, reactions were initiated by addition of NADPH to a final concentration of 2.0 mM and incubated for 15 min. Parallel incubations lacking NADPH were included as negative controls to assess non-enzymatic degradation. Final substrate concentrations were 1.0 μM for both IV-EVE and conventional everolimus. Recombinant enzyme concentrations varied according to the individual CYP isoform and ranged from 5 to 150 nM. CYP1A2, CYP2B6, CYP2C8, CYP2C9, CYP2C19, CYP2D6, and CYP3A4 were evaluated at final concentrations of 100, 150, 40, 20, 80, 5, and 50 nM, respectively. All incubations were performed in triplicate. Reactions were terminated by addition of acetonitrile containing tolbutamide and buspirone as internal standards, followed by centrifugation and LC-MS/MS analysis. To verify enzymatic activity, CYP-selective probe substrates were evaluated in parallel. Phenacetin, bupropion, paclitaxel, diclofenac, mephenytoin, dextromethorphan, and testosterone were used as probe substrates for CYP1A2, CYP2B6, CYP2C8, CYP2C9, CYP2C19, CYP2D6, and CYP3A4, respectively. Formation of the corresponding metabolites in the presence of NADPH confirmed the functional activity of each recombinant enzyme system. Chemical inhibition assay was conducted using pooled human liver microsomes. Human liver microsomes were diluted from a stock concentration of 20 mg/mL to a final incubation concentration of 0.5 mg/mL. Incubations were performed in PBS containing 2.0 mM NADPH at 37 °C for 15 min. Both IV-EVE and conventional everolimus were evaluated at a final concentration of 1.0 μM. Selective CYP inhibitors were used at concentrations previously demonstrated to produce isoform-specific inhibition. α-Naphthoflavone (3 μM), thio-TEPA (20 μM), montelukast (2 μM), sulfaphenazole (20 μM), (±)-N-3-benzylnirvanol (5 μM), quinidine (1 μM), and ketoconazole (1 μM) were employed to inhibit CYP1A2, CYP2B6, CYP2C8, CYP2C9, CYP2C19, CYP2D6, and CYP3A4, respectively. Microsomes and inhibitors were pre-incubated for 5 min at 37 °C prior to addition of substrate and NADPH. Control incubations were conducted in the absence of inhibitors and in the absence of NADPH. All incubations were performed in triplicate. Probe substrate controls corresponding to each CYP isoform were included to verify inhibitor performance. Phenacetin, bupropion, paclitaxel, diclofenac, mephenytoin, dextromethorphan, and testosterone served as positive controls for CYP1A2, CYP2B6, CYP2C8, CYP2C9, CYP2C19, CYP2D6, and CYP3A4, respectively. Following incubation, reactions were terminated by addition of acetonitrile containing internal standards and processed for LC-MS/MS analysis. Everolimus concentrations were quantified by LC-MS/MS using analyte-to-internal standard peak area ratios. In recombinant enzyme studies, amount of parent compound remaining was calculated by comparing incubations conducted in the presence and absence of NADPH. In chemical inhibition studies, the percentage of parent compound remaining and the metabolic contribution of individual CYP isoforms were determined by comparing metabolism in inhibitor-treated incubations with uninhibited controls. CYP contribution was calculated from the reduction in metabolic turnover produced by selective inhibition of individual CYP enzymes. All incubations were performed in triplicate, and results were expressed as mean values with corresponding standard deviations.

### 2.6. Pharmacokinetic and Tissue Distribution Studies

All in vivo studies were conducted at a GLP-certified contract development and manufacturing organization (Shanghai, China) in accordance with institutional standard operating procedures and relevant regulatory guidance. The study protocols were reviewed and approved by the Institutional Animal Care and Use Committee (IACUC) before animal receipt or transfer. Male and female Sprague-Dawley rats (6–8 weeks of age; 179.5–235.7 g) were obtained from Zhejiang Vital River Laboratory Animal Technology Co., Ltd. (Beijing, China) and acclimated prior to study initiation. A total of 8 animals (4 males and 4 females) were assigned to two treatment groups, with each group consisting of two males and two females. The study was conducted to compare the pharmacokinetic profiles of single IV-EVE (3 mg/kg) with everolimus powder formulated in PEG400 vehicle for oral delivery (3 mg/kg) (Oral-EVE). Animals receiving Oral-EVE were fasted overnight before dosing, and food was returned 2 h after administration. Animals receiving IV-EVE remained under the non-fed study conditions. All administrations were performed under yellow-light conditions to minimize potential photodegradation of everolimus. Serial blood samples were collected from each animal for pharmacokinetic analysis. For the IV-EVE, samples were collected pre-dose and at 0.083, 0.25, 0.5, 1, 2, 4, 8, 10, 24, 36, and 48 h following administration. For Oral-EVE, samples were collected pre-dose and at 0.25, 0.5, 1, 2, 4, 8, 10, 24, 36, and 48 h post-dose. Whole blood (0.2 mL) was collected into K2EDTA tubes preloaded with 800 µL acetonitrile. Samples were vortex mixed immediately, maintained on wet ice, and centrifuged at 3200× *g* for 10 min at 2–8 °C. The resulting whole-blood supernatant was harvested and stored at −60 °C to −90 °C until analysis. Everolimus concentrations were quantified using a validated LC–MS/MS assay. The assay lower limit of quantification was 0.3 ng/mL. Quality control samples were included in each analytical batch, and acceptance criteria required that at least 50% of quality control samples at each concentration level and at least two-thirds of all quality control samples fall within 85–115% of nominal concentrations. Pharmacokinetic parameters were calculated using noncompartmental analysis in Phoenix WinNonlin version 8.5 (Certara, Princeton, NJ, USA). Parameters included maximum observed concentration (C_max_), time to maximum concentration (T_max_), area under the concentration–time curve from time zero to the last measurable concentration (AUC_0–t_), area under the curve extrapolated to infinity (AUC_0–∞_), terminal elimination half-life (t½), clearance (CL or CL/F), apparent volume of distribution (Vd or Vd/F), mean residence time (MRT), and initial concentration (C0) where applicable. Concentrations below the lower limit of quantification prior to C_max_ were treated as zero, whereas post-C_max_ values below quantification were excluded from pharmacokinetic calculations.

Dose-proportional Pharmacokinetic Study in Sprague-Dawley Rats. Eighteen SPF-grade Sprague-Dawley rats, consisting of 9 males and 9 females, were assigned to three dose groups with 3 males and 3 females per group. Animals were 6–8 weeks of age at study initiation and weighed 202.6–219.7 g. Animals were not fasted during the study. IV-EVE was administered once by intravenous injection at dose levels of 5, 10, or 15 mg/kg using a dose volume of 5 mL/kg. Administration was performed under yellow-light conditions. Whole blood samples were collected and processed as above. Pharmacokinetic parameters were calculated from whole blood concentration–time data by noncompartmental analysis using Phoenix WinNonlin version 8.5. Predose concentrations below the lower limit of quantitation were excluded, and post-T_max_ BLQ values were not included in the pharmacokinetic analysis. The primary pharmacokinetic parameters included C0, T_max_, C_max_, AUC_0–t_, AUC_0–∞_, terminal half-life, clearance, apparent volume of distribution, mean residence time, and steady-state volume of distribution.

Tissue distribution and excretion of IV-EVE (intravenous) and Oral-EVE (oral with everolimus powder formulated in PEG400 vehicle) were evaluated in rats following a single administration at 3 mg/kg. Sixteen rats (8 males and 8 females) were randomized into eight groups for tissue distribution (*n* = 2 per time point; 1 male and 1 female). Whole-blood and tissue samples were collected at 0.083, 0.5, 8, and 24 h post-dose. Tissues harvested included bone marrow, brain, heart, lungs, liver, stomach, spleen, kidneys, skeletal muscle, abdominal fat, testes, ovaries, uterus, bladder, small intestine, and large intestine. Sixteen rats (8 males and 8 females) were randomized into four groups for excretion (*n* = 4/group; 2 males and 2 females per group) and were housed in metabolic cages for quantitative collection of urine and feces up to 48 h post-dose. Bile samples were collected up to 48 h after dosing.

### 2.7. Toxicological Evaluation

A Good Laboratory Practice (GLP)-compliant repeated-dose toxicity study was conducted at Medicilon Preclinical Research (Shanghai, China) LLC in accordance with National Medical Products Administration (NMPA) guidelines for repeated-dose toxicity and toxicokinetic studies, as well as International Council for Harmonisation (ICH) guidelines M3 (R2), S3A, and S9. The objective of the study was to characterize the toxicity profile, dose–response relationship, target organs, reversibility of findings, and toxicokinetic behavior of IV-EVE following repeated intravenous administration in rats.

A total of 160 Sprague-Dawley rats (80 males and 80 females) were enrolled. Animals were randomized by body weight and sex. Animals were assigned to vehicle control, 5 mg/kg, 10 mg/kg, or 15 mg/kg treatment groups, with 15 animals per sex per group. Male body weights at study initiation ranged from 205.8 to 227.9 g, whereas female body weights ranged from 175.4 to 210.2 g.

IV-EVE was administered by intravenous injection once weekly on Days 1, 8, 15, 22, and 29 at dose levels of 5, 10, or 15 mg/kg. Vehicle-control animals received the formulation vehicle only. The dosing volume was maintained at 10 mL/kg for all groups. Following completion of the dosing phase, animals were observed during a 4-week recovery period to evaluate the reversibility or persistence of treatment-related findings.

Animals underwent an acclimation period prior to initiation of dosing. During acclimation, cage-side observations were performed twice daily. Physical examinations, detailed clinical observations, body weight measurements, food consumption assessments, and ophthalmic examinations were conducted before dosing initiation. During both the treatment and recovery periods, animals were observed twice daily for mortality and clinical signs of toxicity. Detailed clinical examinations, body weight determinations, and food consumption measurements were conducted weekly throughout the study. Ophthalmologic examinations were performed at the end of the dosing period and again at the end of the recovery phase.

Comprehensive clinical pathology evaluations were performed at the conclusion of both the dosing and recovery phases. Blood samples were collected for hematology, coagulation, and serum chemistry analyses, while urine samples were obtained for urinalysis evaluation. Hematology assessments included erythrocyte, leukocyte, and platelet parameters. Coagulation analyses included measurements of clotting function and fibrinogen concentrations. Clinical chemistry evaluations assessed hepatic, renal, metabolic, and electrolyte parameters. Urinalysis included standard assessments of urine composition and renal function indicators. These evaluations were conducted on all surviving animals designated for terminal necropsy at the end of the dosing period and following the recovery phase.

All animals surviving to scheduled termination underwent complete gross necropsy examinations at the end of either the dosing phase or recovery phase. The following organs were collected: central nervous system (brain, spinal cord, sciatic nerve), lymphoid and hematopoietic system (thymus, spleen, mandibular lymph node, mesenteric lymph node, bone marrow (sternum/femur)), cardiovascular system (heart, aorta, respiratory system, lung, trachea, nasal cavity), gastrointestinal system (tongue, esophagus, stomach, forestomach, glandular stomach, duodenum, jejunum, ileum, cecum, colon, rectum, salivary glands), hepatobiliary and pancreatic system (liver, gallbladder, pancreas), urinary system (kidneys, urinary bladder, endocrine system, pituitary, thyroid, parathyroid, adrenal glands), reproductive system—male (testes, epididymides, prostate, seminal vesicles, coagulating glands), reproductive system—female (ovaries, uterus, cervix, vagina, mammary gland), musculoskeletal system (skeletal muscle, bone (femur/sternum), special sense organs (eyes, optic nerve, harderian gland), integumentary system (skin), other (injection site, any gross lesions). Organs were weighed, and organ-to-body-weight and organ-to-brain-weight ratios were calculated where appropriate. Bilateral organs were weighed together. Gross pathological findings were recorded and correlated with clinical observations and clinical pathology results. The external surface and all major body cavities, including the cranial, thoracic, abdominal, and pelvic cavities, were systematically examined for macroscopic abnormalities. Any gross lesions identified during necropsy were recorded and collected for subsequent histopathological evaluation.

Following collection, tissues were fixed in appropriate fixatives according to standard GLP toxicologic pathology procedures. Most tissues were preserved in 10% neutral buffered formalin, while selected tissues requiring specialized fixation, including ocular and male reproductive tissues, were processed using tissue-appropriate fixatives before routine histologic processing. After fixation, tissues were dehydrated through graded alcohols, cleared, embedded in paraffin, sectioned at approximately 3–5 μm thickness, mounted on glass slides, and stained with hematoxylin and eosin (H&E) for microscopic evaluation. Histopathological examinations were conducted by a board-certified veterinary pathologist. Microscopic evaluation was initially performed on tissues collected from control and high-dose animals. Following identification of treatment-related findings, the corresponding tissues from lower-dose groups were examined to establish dose–response relationships. Lesions were characterized and graded according to standard toxicologic pathology criteria. Animals assigned to recovery groups were evaluated separately to assess the reversibility, persistence, or progression of treatment-related findings following cessation of dosing.

Assessment of gastrointestinal toxicity was based on direct histopathological examination of tissue architecture and morphology. Gastrointestinal tissues, including the stomach, duodenum, jejunum, ileum, cecum, colon, and rectum, were examined for evidence of inflammation, epithelial hyperplasia, degeneration, ulceration, necrosis, or other treatment-related lesions. Because toxicity determinations were based on microscopic pathology rather than tissue drug content measurements, the evaluation reflected tissue injury rather than residual luminal drug. Similarly, oral and upper gastrointestinal tissues, including the tongue, salivary glands, and esophagus, were examined as part of the routine histopathology assessment.

Continuous variables, including body weight, food consumption, clinical pathology parameters, toxicokinetic measurements, and organ weight data, were summarized using descriptive statistics. Comparisons between treated and concurrent vehicle-control groups were performed separately by sex. Statistical significance was evaluated using appropriate parametric or non-parametric methods based on data distribution and variance characteristics. Toxicological interpretation incorporated statistical significance, magnitude of change, dose–response relationship, biological relevance, and correlation with pathological findings to determine treatment-related effects and adverse event thresholds.

The statistical analysis for body weight, clinical chemistry parameters, hematology (not including leukocyte counts), coagulation measures, and organ weights was analyzed using group pairwise comparisons (Levene’s + ANOVA + Dunnett/Welch + Bonferroni/Welch). Urinalysis was evaluated using rank transformation/group pairwise comparisons (rank + Kruskal–Wallis + Dunnett). Leukocyte count was analyzed by log transformation/group pairwise comparisons (log/Levene’s + ANOVA + Dunnett/Welch + Bonferroni/Welch).

This study included evaluation of both immediate toxicological effects and recovery following cessation of treatment, enabling determination of target organs, reversibility of treatment-related findings, and establishment of exposure–response relationships for IV-EVE.

### 2.8. Bioanalytical Analysis

EVE concentrations in whole blood, tissue, urine, feces, and bile in rats were determined using a validated LC–MS/MS method with positive electrospray ionization (ESI) in multiple reaction monitoring (MRM) mode. Rapamycin was used as the internal standard (IS) in all assays. Chromatographic separation was achieved on an Agilent ZORBAX SB-CN column (3.5 μm, 100 × 2.1 mm) maintained at 40 °C, with an autosampler temperature of 4 °C. The mobile phases consisted of (A) 0.3% formic acid with 2 mM ammonium formate in water and (B) acetonitrile, delivered under gradient elution. Detection was performed on a Triple Quad 6500+ mass spectrometer (Sciex, Marlborough, MA, USA) with MRM transitions *m*/*z* 975.50 → 908.40 for EVE and *m*/*z* 931.50 → 864.50 for rapamycin. For whole blood matrices, K_2_EDTA-anticoagulated samples (40 μL) were processed, and quantification was based on analyte-to-IS peak area ratios over a calibration range of 1.00–1000.00 ng/mL (LLOQ 1.00 ng/mL; ULOQ 1000.00 ng/mL). For rat liver tissue, urine, and feces, the EVE reference standard and rapamycin internal standard were prepared in DMSO at 2 mg mL^−1^ and 1 mg mL^−1^, respectively. Matrix-matched calibration curves covered 1.00–1000.00 ng mL^−1^ for liver tissue, 0.300–300.000 ng mL^−1^ for urine, 0.500–500.000 ng mL^−1^ for feces, and 1.00–1000.00 ng mL^−1^ for bile; coefficients of determination were ≥0.9952 for liver and feces and ≥0.9978 for bile, meeting acceptance criteria of r^2^ ≥ 0.9800. Samples (20 µL) were mixed with 400 µL of IS working solution in methanol, vortex-mixed, centrifuged (≈4000 rpm, 10 min, 4 °C), and 300 µL of supernatant was injected for LC-MS/MS analysis; blank and zero-blank controls were processed in parallel. System suitability required peak area ratio RSD ≤ 15% and retention time RSD ≤ 5%, criteria that were met in all matrices. Accuracy and precision satisfied ±15% (±20% at LLOQ) in intra- and inter-day assessments across all QC levels.

For the tissue distribution study, after collection, each tissue sample—including stomach, small intestine, and large intestine—was rinsed separately with normal saline to prevent cross-contamination, blotted dry with filter paper, placed into an individually labeled tube, sealed, weighed, and stored at −60 °C to −90 °C until LC-MS/MS analysis.

## 3. Results

### 3.1. LC/MS Method Validation

#### 3.1.1. Bioanalytical Method Validation for Quantification of Everolimus in Rat Whole Blood

Everolimus concentrations in K2EDTA-anticoagulated whole blood from Sprague-Dawley rats were quantified using a validated liquid chromatography–tandem mass spectrometry (LC–MS/MS) method developed and validated in accordance with the Chinese Pharmacopoeia (2020), U.S. Food and Drug Administration Bioanalytical Method Validation Guidance (2018), and ICH M10 Bioanalytical Method Validation and Study Sample Analysis Guideline (2022). The assay was validated over a calibration range of 1.0–1000 ng/mL to support pharmacokinetic and toxicokinetic investigations.

Blank whole blood obtained from Sprague-Dawley rats containing K2EDTA anticoagulant was used as the biological matrix for preparation of calibration standards and quality control (QC) samples. Calibration standards spanning the analytical range were prepared in matrix together with lower limit of quantification (LLOQ), low (LQC), geometric middle (GMQC), middle (MQC), and high (HQC) QC samples. Samples exceeding the upper limit of quantification were evaluated through validated dilution procedures using 10-fold and 100-fold dilutions.

Quantitative analysis was performed by LC–MS/MS using an internal-standard-based approach. Method validation included assessment of system suitability, selectivity, carryover, calibration curve performance, intra- and inter-assay accuracy and precision, dilution integrity, extraction recovery, matrix effects, and stability under anticipated sample handling and storage conditions. Recovery and matrix effect evaluations were performed at low and high QC concentrations using six independent matrix lots. IS-normalized matrix factors were calculated to assess ion suppression or enhancement.

Selectivity was assessed using individual blank matrix lots to demonstrate the absence of endogenous interferences at the retention times of everolimus and the internal standard. Carryover was evaluated by analysis of blank samples following upper-limit calibration standards. Calibration curves were accepted when at least 75% of calibration levels met predefined acceptance criteria, including acceptable performance at both the LLOQ and ULOQ, with a minimum coefficient of determination (r^2^) of 0.98. Accuracy and precision were evaluated across multiple analytical runs using replicate QC samples, with acceptance criteria consistent with regulatory guidance.

LC–MS/MS method was established for quantification of everolimus in K2EDTA whole blood from Sprague-Dawley rats over a concentration range of 1.0–1000 ng/mL. The assay demonstrated robust chromatographic performance, with excellent system suitability characterized by highly reproducible retention times and peak area ratios between everolimus and the internal standard. Relative standard deviations (RSDs) for peak area ratios were ≤2.55% in rat whole blood and ≤2.01% in dog whole blood, while retention time variability remained below 0.13% for all analyses.

No endogenous matrix interferences were detected at the retention times corresponding to everolimus or the internal standard in any of the six independent matrix lots evaluated. Likewise, no significant analyte-to-internal standard or internal standard-to-analyte interference was observed. Carryover was negligible, with blank samples following upper-limit calibrators demonstrating no detectable analyte or internal standard signal above predefined acceptance criteria. These findings confirmed the high selectivity and specificity of the assay.

Calibration curves demonstrated excellent linearity throughout the validated range. In rat whole blood, coefficients of determination (r^2^) were ≥0.9972. Relative errors for calibration standards remained well within regulatory acceptance criteria, with LLOQ samples ranging from −7.41% to 8.88% in rats and −7.34% to 7.28% in dogs. Across all non-LLOQ calibration levels, relative errors remained within approximately ±10%, confirming excellent quantitative performance across the analytical range.

Intra- and inter-assay accuracy and precision met all regulatory requirements. For rat whole blood, intra-assay precision across QC levels was ≤4.88% and ≤6.42% at the LLOQ, while inter-assay precision was ≤3.88% across QC levels and 7.14% at the LLOQ. Mean inter-assay accuracy ranged from −0.22% to 2.61% across QC concentrations.

Ancillary validation QC samples further confirmed assay robustness, with all measured concentrations remaining within ±15% of nominal values in both species. Dilution integrity studies demonstrated accurate quantification of samples exceeding the upper limit of calibration following both 10-fold and 100-fold dilution. Mean relative errors after dilution ranged from 9.17% to 9.53% in rat whole blood, with all diluted samples satisfying acceptance criteria.

Extraction recovery of everolimus and the internal standard was consistent across all evaluated concentration levels. Recovery variability remained low, with RSD values ≤6.21% in rat matrices. Matrix effect evaluations demonstrated minimal ion suppression or enhancement, as evidenced by IS-normalized matrix factor variability ≤3.84% in rat matrix. Quantitative matrix effect assessments across six independent lots showed mean relative errors ranging from −2.00% to 5.11% in rat whole blood, confirming that matrix composition did not significantly affect analyte quantification.

Comprehensive stability studies demonstrated that everolimus remained stable throughout sample collection, processing, storage, and analytical procedures. In rat whole blood, analyte stock and working solutions remained stable during short-term bench handling and extended frozen storage, while whole blood samples maintained 94.6–102.5% of baseline signal after 2 h under both room-temperature and wet-ice conditions. Long-term frozen storage, freeze–thaw cycling, processed sample storage, autosampler residence, and reinjection conditions all met predefined acceptance criteria. Collectively, these data demonstrate that the LC–MS/MS assay provided a sensitive, selective, accurate, and reproducible method for quantification of everolimus in rat whole blood. The validated assay supported subsequent pharmacokinetic, tissue distribution, and toxicokinetic investigations by enabling reliable measurement of everolimus concentrations across a broad analytical range with demonstrated stability under all relevant handling and storage conditions.

#### 3.1.2. Bioanalytical Analysis of Everolimus in Tissue and Excreta Samples

In addition to whole blood analysis, separate matrix-specific LC–MS/MS methods were developed and partially validated for quantification of everolimus in Sprague-Dawley rat liver tissue, urine, feces, and bile. Because these matrices differ substantially in composition, protein content, endogenous lipid burden, and potential ion-suppression characteristics, partial validations were performed using matrix-matched calibration standards and quality control samples prepared in each individual biological matrix.

The whole-blood assay represented the primary fully validated bioanalytical method and utilized K2EDTA-anticoagulated blood with a calibration range of 1–1000 ng/mL. In contrast, tissue and excreta analyses required matrix-specific sample preparation procedures prior to LC–MS/MS analysis. Liver and fecal samples were first homogenized to generate uniform analytical matrices suitable for extraction and quantification, whereas urine and bile samples were analyzed as liquid matrices following appropriate dilution and extraction procedures. Matrix-specific calibration standards, quality control samples, recovery assessments, and matrix-effect evaluations were prepared independently for each biological matrix to account for differences in extraction efficiency and ionization behavior.

The liver tissue assay was partially validated over a concentration range of 1.0–1000 ng/mL, matching the analytical range used for whole blood. Liver tissue presents a substantially more complex matrix than blood because of high lipid content, intracellular proteins, and tissue-associated phospholipids that can contribute to ion suppression during mass spectrometric detection. Consequently, matrix-specific recovery, dilution integrity, and matrix-effect evaluations were performed using homogenized liver tissue preparations.

Urine samples were analyzed using a more sensitive assay with a validated concentration range of 0.3–300 ng/mL. Because urine contains relatively low protein content but highly variable salt concentrations and endogenous metabolites, separate validation of matrix effects, recovery, dilution integrity, and analyte stability was required. The lower calibration range was selected to support quantification of the expected low urinary excretion of everolimus and its limited renal elimination.

Fecal samples were analyzed following homogenization of fecal material and extraction of everolimus from the resulting suspension. The fecal assay was validated over a concentration range of 0.5–500 ng/mL. Compared with blood, fecal matrices contain substantial particulate matter, dietary residues, microbial biomass, bile-derived lipids, and endogenous metabolites that can significantly affect analyte recovery and ionization efficiency. Therefore, separate evaluations of extraction recovery, matrix effects, dilution integrity, and stability were conducted using matrix-matched fecal controls.

Bile samples were analyzed using a partially validated assay covering 1.0–1000 ng/mL. Bile represents a particularly challenging matrix because of its high concentrations of bile salts, phospholipids, cholesterol, and endogenous lipophilic compounds that may influence extraction efficiency and electrospray ionization. As with the other matrices, calibration standards and quality controls were prepared in blank bile matrix, and matrix-specific validation was conducted to establish assay selectivity, accuracy, precision, recovery, dilution integrity, and stability.

For all matrices, LC–MS/MS validation included assessment of system suitability, selectivity, carryover, calibration curve performance, intra-assay accuracy and precision, dilution integrity, extraction recovery, matrix effects, and analyte stability under anticipated handling and storage conditions. Partial validation was considered appropriate because the chromatographic and mass spectrometric platform had previously undergone full validation in whole blood, while the additional studies were designed specifically to demonstrate suitability of the method in alternate biological matrices used for tissue distribution and excretion analyses.

Despite the substantially different physicochemical characteristics of these matrices, all assays demonstrated acceptable selectivity, sensitivity, accuracy, precision, recovery, matrix-effect control, dilution integrity, and stability, supporting their suitability for quantitative bioanalysis in ADME investigations.

System suitability testing demonstrated highly reproducible chromatographic performance across all matrices. The relative standard deviation of analyte-to-internal standard peak area ratios remained below 2.8% in all assays, while retention time variability was consistently less than 0.15%, confirming stable chromatographic performance throughout validation.

No endogenous interference was observed at the retention times corresponding to everolimus or the internal standard in any matrix. Selectivity assessments demonstrated the absence of matrix-derived peaks that could affect analyte quantification, and carryover remained below predefined acceptance criteria for all methods. These findings confirmed assay specificity despite the complexity of liver tissue, bile, and fecal matrices.

### 3.2. Metabolism

**Figure 1:**
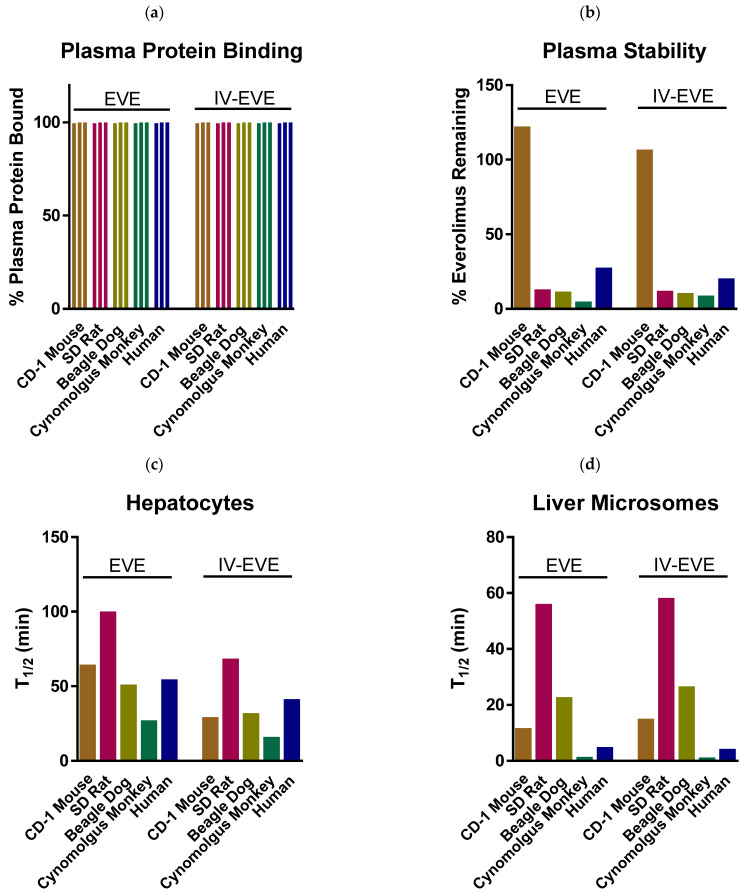

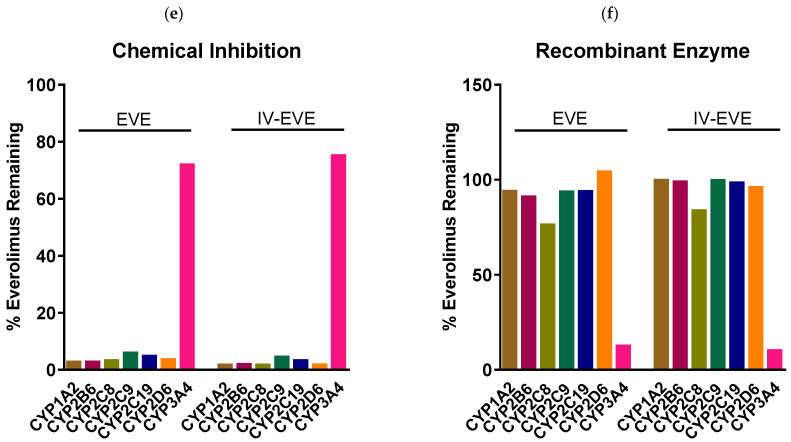
IV-EVE and EVE showed similar metabolism across 5 different species, including mouse, rat, dog, monkey, and human. (**a**) Percentage of plasma protein binding of 0.2 to 2 µM (left to right in each group) EVE for 10 min incubation. (**b**) Percentage of EVE remaining after 120 min incubation in plasma stability. (**c**) Half-life of EVE in hepatocyte stability. (**d**) Half-life of EVE in liver microsomal stability. (**e**) Percentage of EVE remaining after incubating the inhibitors of enzymes (CYP1A2, CYP2B6, CYP2C8, CYP2C9, CYP2C19, CYP2D6 and CYP3A4) for 15 min. (**f**) Percentage of EVE remaining after incubating with recombinant enzymes (CYP1A2, CYP2B6, CYP2C8, CYP2C9, CYP2C19, CYP2D6 and CYP3A4) for 15 min.

EVE metabolism was unchanged, going from unformulated EVE to formulated IV-EVE. Plasma binding and plasma degradation remained the same. Upon incubation with plasma, more than 99.6% of IV-EVE and 99.5% of EVE became protein-bound at 0.2–20 µM across five different species, including mouse, rat, dog, monkey, and human (Figure 1a). Plasma stability was evaluated across five different species, including mouse, rat, dog, monkey, and human. Both IV-EVE and EVE groups showed similar remaining levels after 120 min of incubation (Figure 1b). EVE was actively metabolized by both hepatocytes and liver microsomes; both EVE and IV-EVE showed comparable in vitro metabolic profiles in hepatocytes and liver microsomes in the same species (Figure 1c,d). CYP3A4 remained the most active metabolizer with minimal contribution from the remaining CYP isoforms. On incubation with microsomes in the presence of specific CYP inhibitors, the CYP3A4 inhibitor was the most effective (Figure 1e). Recombinant enzyme assays demonstrated comparable metabolic susceptibility between IV-EVE and conventional everolimus, with similar residual parent fractions after CYP3A4 incubation (Figure 1f).

Across all species examined, IV-EVE and conventional everolimus displayed highly comparable plasma stability profiles. The half-lives observed for IV-EVE versus conventional everolimus were 41.6 versus 41.7 min in rat plasma, 39.5 versus 36.7 min in dog plasma, 34.3 versus 28.1 min in monkey plasma, and 53.8 versus 64.4 min in human plasma. These findings indicate that IV-EVE did not substantially alter the intrinsic plasma metabolic stability of the active pharmaceutical ingredient.

The metabolic stability of IV-EVE and conventional everolimus was evaluated in liver microsomes derived from mouse, rat, dog, monkey, and human liver tissues. Across all species, both formulations underwent rapid NADPH-dependent metabolism, consistent with extensive hepatic CYP-mediated biotransformation of everolimus. In mouse liver microsomes, IV-EVE exhibited a metabolic half-life of 15.0 min compared with 11.7 min for conventional everolimus, corresponding to predicted hepatic extraction ratios of 80.2% and 83.8%, respectively. Similar findings were observed in dog microsomes, where IV-EVE demonstrated a half-life of 26.6 min compared with 22.8 min for conventional everolimus. In human microsomes, IV-EVE exhibited a half-life of 4.22 min versus 4.90 min for conventional everolimus, indicating essentially equivalent susceptibility to oxidative metabolism. Cynomolgus monkey microsomes showed the most rapid turnover for both formulations, with half-lives of 1.19 min and 1.44 min for IV-EVE and conventional everolimus, respectively. In contrast, rat microsomes exhibited substantially slower metabolism, with half-lives of 58.2 min for IV-EVE and 56.1 min for conventional everolimus. Predicted intrinsic clearance values followed similar trends. IV-EVE exhibited intrinsic clearance values ranging from 42.9 to 1571 mL/min/kg across species, whereas conventional everolimus ranged from 44.4 to 1303 mL/min/kg. Both formulations were classified as high-clearance compounds in mouse, dog, monkey, and human microsomes and moderate-clearance compounds in rat microsomes. Overall, the microsomal data demonstrated that IV-EVE did not materially alter susceptibility to hepatic CYP-mediated metabolism, indicating that the active pharmaceutical ingredient remained available for normal metabolic processing following release from the formulation.

To assess metabolism in a more physiologically relevant cellular system, metabolic stability was evaluated in cryopreserved hepatocytes containing intact phase I and phase II metabolic pathways. Similar to the microsomal findings, both IV-EVE and conventional everolimus underwent substantial metabolism across all species examined. In CD-1 mouse hepatocytes, IV-EVE exhibited a half-life of 29.3 min compared with 64.6 min for conventional everolimus. In Sprague-Dawley rat hepatocytes, the corresponding half-lives were 68.5 min and 100 min. Beagle dog hepatocytes yielded half-lives of 31.9 min and 51.1 min for IV-EVE and conventional everolimus, respectively. Cynomolgus monkey hepatocytes demonstrated the most rapid metabolism, with half-lives of 16.0 min and 27.2 min, whereas human hepatocytes exhibited half-lives of 41.3 min and 54.6 min for IV-EVE and conventional everolimus, respectively.

Across all CYP isoforms examined, both formulations demonstrated highly similar metabolic profiles, indicating that IV-EVE did not alter the fundamental metabolic pathways responsible for drug clearance. After incubation with recombinant CYP1A2, CYP2B6, CYP2C8, CYP2C9, CYP2C19, and CYP2D6 for 15 min in the presence of NADPH, minimal metabolism of either formulation was observed. Parent compound remaining for IV-EVE ranged from 84.4% to 100.4%, whereas parent remaining for conventional everolimus ranged from 76.9% to 104.8%. The greatest metabolism outside of CYP3A4 was observed with CYP2C8, where 84.4% of IV-EVE and 76.9% of conventional everolimus remained after incubation, suggesting only a minor contribution of this isoform to overall metabolic turnover. In contrast, both formulations underwent extensive metabolism in the presence of recombinant CYP3A4. Following a 15 min incubation, only 10.9% of IV-EVE and 13.3% of conventional everolimus remained relative to corresponding incubations lacking NADPH, representing approximately 89% and 87% parent depletion, respectively. The magnitude of CYP3A4-mediated metabolism was substantially greater than that observed for any other CYP isoform, demonstrating that CYP3A4 is the dominant metabolic pathway for both formulations.

To further define the relative contribution of individual CYP enzymes under physiologically relevant conditions, selective chemical inhibition studies were performed using pooled human liver microsomes. In the absence of inhibitors, both formulations were rapidly metabolized during the 15 min incubation period. Only 1.4% of IV-EVE and 2.4% of conventional everolimus remained relative to control incubations lacking NADPH, corresponding to metabolic turnover of 98.6% and 97.6%, respectively. These findings confirm the extensive susceptibility of everolimus to hepatic oxidative metabolism. Selective inhibition of CYP1A2, CYP2B6, CYP2C8, CYP2C9, CYP2C19, or CYP2D6 produced only modest increases in parent compound remaining for either formulation. Calculated contributions of these enzymes to overall metabolism were uniformly low, ranging from 0.8% to 3.6% for IV-EVE and from 0.7% to 4.1% for conventional everolimus. In contrast, inhibition of CYP3A4 with ketoconazole produced a profound reduction in metabolic turnover. Parent compound remaining increased from 1.4% to 75.6% for IV-EVE and from 2.4% to 72.4% for conventional everolimus. Correspondingly, metabolic turnover decreased from 98.6% to 24.4% for IV-EVE and from 97.6% to 27.6% for conventional everolimus. Calculated CYP3A4 contributions were 75.3% and 71.7% for IV-EVE and conventional everolimus, respectively, substantially exceeding contributions from all other CYP isoforms.

### 3.3. Excretion

Very little intact EVE was excreted with excretion similar for Oral-EVE versus IV-EVE. In rats, parent drug recovery over 48 h following IV-EVE (3 mg/kg) was minimal (Table 1). Similar low parent drug recovery was observed after oral EVE treatment. Overall, elimination occurred predominantly via metabolic biliary/fecal clearance, with negligible renal excretion of unchanged drug.

### 3.4. Distribution

**Figure 2:**
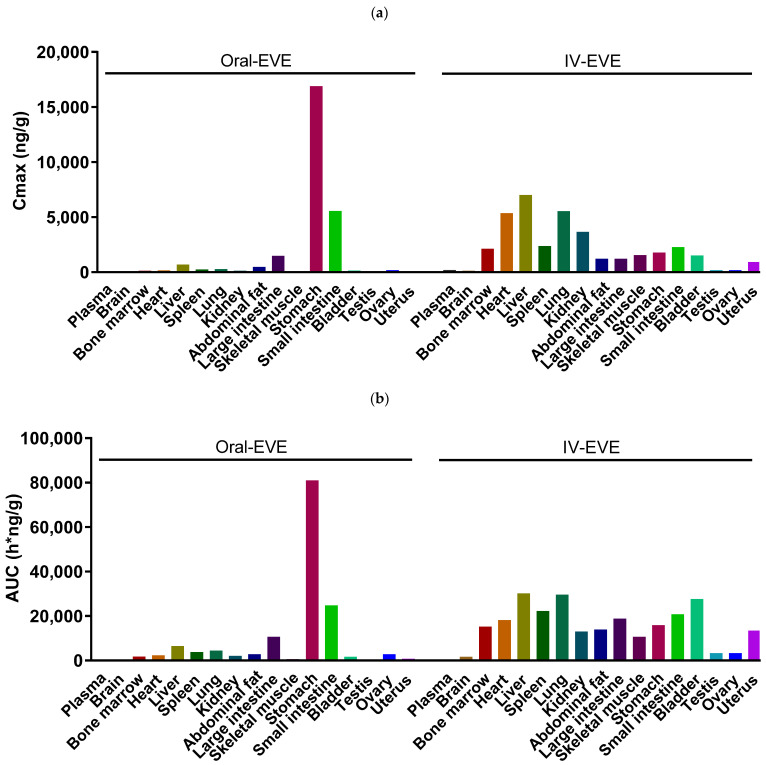
IV-EVE exhibited rapid and uniform distribution, whereas EVE was preferentially found in the GI tract. (**a**) C_max_ and (**b**) AUC of organ distribution profile for Oral-EVE and IV-EVE.

Distribution was affected by changing from Oral-EVE to IV-EVE. Following 3 mg/kg of Oral-EVE or IV-EVE administration in rats, Oral-EVE was mainly found in the stomach and intestines (Figure 2). In contrast, IV-EVE exhibited rapid and extensive distribution across all tissues after administration (Figure 2). In addition, tissue-to-plasma AUC ratios were high in liver, lung, bladder, spleen, and small intestine tissues, with lower penetration into the brain, with a minimal amount of EVE detected in plasma or blood.

### 3.5. Absorption

**Figure 3:**
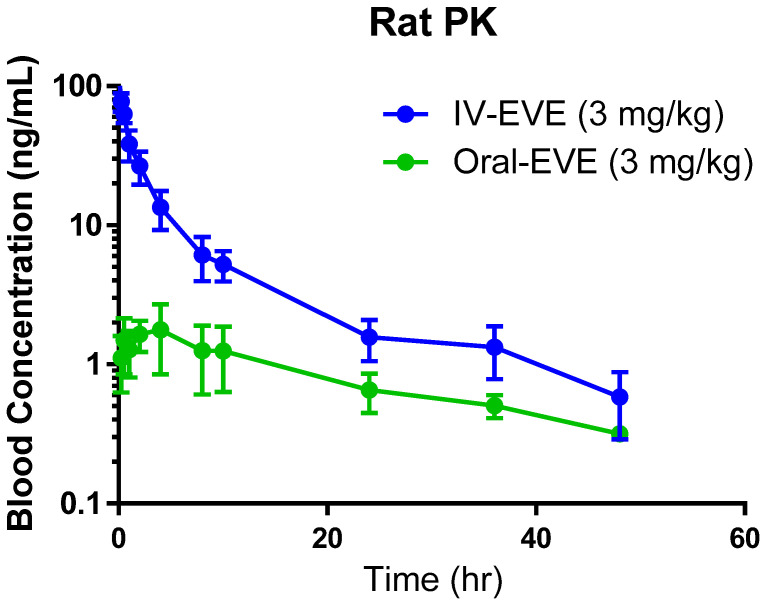
Blood PK for IV-EVE was higher than blood PK for Oral-EVE in rats. Bolus administration of IV-EVE is characterized by C_max_ that is 100× higher than Oral-EVE.

In rats, a single 3 mg/kg IV bolus injection produced robust systemic exposure (Figure 3). In males, C_max_ was 133.448 ng/mL, with an AUC_0–∞_ of 339.203 h·ng/mL; in females, C_max_ was 98.195 ng/mL, with an AUC_0–∞_ of 220.930 h·ng/mL (Table 2). The terminal half-life (t½) was comparable between sexes (12.88 h in males; 12.67 h in females). There was no difference in T1/2 between Oral-EVE and IV-EVE; AUC_0–t_ increased by 7–9×, and C_max_ increased by 54–55×.

IV-EVE exhibited dose-proportional PK. Across the 5–15 mg/kg range, both C_max_ and AUC increased proportionally with dose (Table 3). Following a single intravenous administration of IV-EVE, everolimus was rapidly detected in whole blood at the first sampling time point (0.083 h) in all dose groups. Peak concentrations occurred at the earliest measured time point (T_max_ = 0.083 h) for both male and female animals, consistent with immediate systemic delivery following intravenous administration.

Whole-blood concentration–time profiles demonstrated a biphasic decline characterized by a rapid distribution phase followed by a prolonged elimination phase extending through the 48 h observation period. Quantifiable concentrations remained detectable at the final sampling time point in most animals, indicating sustained systemic exposure after a single administration. Systemic exposure increased with increasing dose across the evaluated range of 5–15 mg/kg. In male rats, mean C_max_ increased from 330 ± 7 ng/mL at 5 mg/kg to 737 ± 33 ng/mL at 10 mg/kg and 900 ± 88 ng/mL at 15 mg/kg. Corresponding AUC_0–t_ values increased from 723 ± 78 h·ng/mL to 1665 ± 203 h·ng/mL and 2377 ± 385 h·ng/mL, respectively. Similar trends were observed in female rats, where mean C_max_ values increased from 300 ± 3 ng/mL to 632 ± 83 ng/mL and 1103 ± 220 ng/mL, while AUC_0–t_ increased from 632 ± 178 h·ng/mL to 1412 ± 176 h·ng/mL and 2457 ± 244 h·ng/mL across the same dose levels.

Assessment of dose proportionality demonstrated that increases in systemic exposure closely tracked dose escalation. Relative dose ratios of 1:2:3 produced corresponding AUC_0–t_ ratios of approximately 1:2.30:3.29 in males and 1:2.23:3.89 in females. Similarly, C_max_ ratios increased approximately proportionally with dose. These findings indicate near-dose-proportional pharmacokinetics over the investigated dose range.

The terminal elimination half-life ranged from approximately 9 to 20 h across dose groups, indicating sustained systemic persistence following intravenous administration. Mean half-life values were 11.4–13.0 h at 5 mg/kg, 13.0–20.0 h at 10 mg/kg, and 8.7–9.1 h at 15 mg/kg. Clearance values remained relatively consistent across dose levels, ranging from approximately 5.9 to 7.9 L/h/kg, while apparent volumes of distribution were substantially greater than total body water, ranging from approximately 78 to 168 L/kg. These findings suggest extensive tissue distribution of everolimus following administration of IV-EVE.

Comparison of pharmacokinetic parameters between male and female animals demonstrated minimal sex-related differences. Male-to-female C_max_ ratios ranged from 0.82 to 1.17 across dose groups, while AUC_0–t_ ratios ranged from 0.97 to 1.18. These differences were not considered biologically meaningful and did not demonstrate a consistent directional trend. Accordingly, systemic exposure to everolimus following administration of IV-EVE appeared comparable between sexes.

### 3.6. Toxicology Evaluation

Survival, Clinical Observations, Ophthalmology, and Injection Site Tolerability: All animals survived to their scheduled necropsy time points, and no treatment-related mortality occurred at any dose level throughout the study. No IV-EVE-related abnormalities were observed during cage-side or detailed clinical observations. Ophthalmologic examinations performed during both the dosing and recovery phases revealed no treatment-related findings. Similarly, no evidence of local intolerance, irritation, or adverse reactions at the intravenous administration site was identified. Urinalysis parameters remained unremarkable and showed no treatment-related alterations. These findings indicate that repeated weekly intravenous administration of IV-EVE was generally well tolerated from a clinical perspective and did not produce overt systemic toxicity, behavioral abnormalities, or local injection site injury.

Body Weight and Food Consumption: The primary in-life toxicity findings consisted of modest reductions in body weight gain and food consumption. Male animals receiving ≥5 mg/kg exhibited reduced body weight gain beginning after the first week of dosing and decreased food consumption during the dosing period. These findings were dose-related but relatively small in magnitude and were not accompanied by clinical signs of illness. Importantly, these effects were reversible. By the end of the 4-week recovery period, food consumption had returned to control values and body weight deficits demonstrated substantial recovery.

Hematology: Treatment-related hematologic changes were observed primarily at the end of the dosing phase. Male and female animals receiving ≥5 mg/kg exhibited increases in erythrocyte count (RBC), hemoglobin concentration (HGB), and hematocrit (HCT), although the increase in RBC was not evident in females at 5 mg/kg. In addition, male animals receiving ≥10 mg/kg demonstrated increased absolute and relative neutrophil counts, while females receiving 15 mg/kg showed increased basophil counts. The magnitude of these changes was modest. The study pathologists concluded that the erythrocyte-related findings were likely secondary to reduced body weight gain and food intake rather than direct bone marrow toxicity. The neutrophil and basophil elevations were interpreted as evidence of a mild inflammatory response. All hematologic findings resolved during the recovery period.

Coagulation Parameters: Treatment-related coagulation changes were characterized by increased fibrinogen concentrations (FBGs) in females at ≥5 mg/kg and in males at ≥10 mg/kg. Males at ≥10 mg/kg also exhibited shortened thrombin time (TT). These findings were considered consistent with a mild inflammatory or pro-coagulant physiological response. However, no thrombotic lesions, clinical sequelae, or evidence of disseminated coagulation abnormalities were identified. Fibrinogen concentrations returned toward normal during recovery.

Clinical Chemistry: Several treatment-related changes were observed in serum chemistry parameters. Male animals receiving 15 mg/kg demonstrated decreased albumin concentrations, whereas globulin concentrations were increased in males at ≥10 mg/kg. Consequently, albumin/globulin ratios were decreased in high-dose males and in females receiving ≥5 mg/kg. Total cholesterol concentrations were increased in both sexes at all dose levels. Alkaline phosphatase activity was elevated in males at ≥5 mg/kg. Female animals at ≥10 mg/kg exhibited decreased creatinine concentrations, while serum sodium concentrations were increased in females receiving ≥5 mg/kg. Collectively, the pattern of increased globulin, reduced albumin/globulin ratio, elevated fibrinogen, and neutrophilia supports the presence of a mild systemic inflammatory response. Increased cholesterol and alkaline phosphatase may suggest subtle alterations in lipid metabolism and hepatobiliary physiology; however, no histopathologic liver injury was reported. All clinical chemistry findings resolved during recovery.

Gross Pathology: No treatment-related gross pathological lesions were identified during necropsy. No gastrointestinal lesions, ulcerations, erosions, distension, hemorrhage, or other macroscopic abnormalities were reported in the stomach or intestines.

Organ Weight Changes: Treatment-related organ weight changes were primarily observed in lymphoid and reproductive tissues. Decreased thymus weights were observed in both sexes. Male animals demonstrated reduced testes and epididymides weights and corresponding organ-to-body weight ratios. These findings correlated with microscopic changes identified during histopathologic examination.

Histopathology (Table 4): Histopathology identified several target organs of toxicity. The thymus demonstrated decreased cortical lymphocyte cellularity, consistent with lymphoid depletion and immunosuppressive activity of mTOR inhibition. These findings largely resolved following the recovery period. The lung demonstrated alveolar foamy macrophage accumulation. Although the incidence and severity decreased during recovery, residual findings remained in a subset of animals at the end of the recovery phase. Treatment-related myocardial necrosis accompanied by inflammatory cell infiltration was observed during the dosing phase. During recovery, some animals exhibited residual myocardial mononuclear cell infiltrates and fibrosis. Male reproductive organs represented another major target. Testes demonstrated germ cell degeneration within seminiferous tubules during dosing. At the end of recovery, degeneration had progressed to seminiferous tubular atrophy in some animals, accompanied by epididymal luminal cell debris and reduced sperm content.

Gastrointestinal Toxicity Assessment: There was no detectable gastrointestinal toxicity despite repeated intravenous administration of everolimus. No treatment-related gastrointestinal clinical signs such as diarrhea, reduced stool output, gastrointestinal bleeding, or abdominal abnormalities were reported. No treatment-related findings were observed in urinalysis or general clinical observations that would suggest gastrointestinal dysfunction. Most importantly, neither gross pathology nor histopathology identified stomach, small intestinal, or large intestinal lesions. The final pathology summary identified the thymus, heart, lung, testes, epididymides, and lymph nodes as target organs, but did not identify any gastrointestinal tissues as targets of toxicity. All animals survived to their scheduled necropsy time points, and no treatment-related mortality occurred at any dose level throughout the study.

## 4. Discussion

The present study aimed to improve upon the pharmacokinetic and formulation limitations of Oral-EVE through the development of an intravenous derivative, IV-EVE. In a single-dose rat pharmacokinetic study, intravenous IV-EVE (3 mg/kg) demonstrated marked pharmacokinetic advantages over oral EVE formulations. Compared with Oral-EVE at the same dose, IV-EVE achieved substantially higher systemic exposure, with mean C_max_ values increased by approximately 55-fold in males (133.448 vs. 2.408 ng/mL) and 54-fold in females (98.195 vs. 1.822 ng/mL), and AUC_(0–∞)_ increased by 6.5-fold (339.203 vs. 52.169 h·ng/mL) in males and 7.4-fold (220.930 vs. 29.483 h·ng/mL) in females. Oral bioavailability was limited 15% for Oral-EVE, whereas intravenous administration ensured complete systemic delivery, eliminating first-pass and absorption-related losses. In addition, IV-EVE provided rapid systemic attainment (T_max_ 0.083 h) compared with delayed oral absorption (T_max_ 1.25–4.00 h), and exhibited lower pharmacokinetic variability than oral formulations, which are inherently subject to gastrointestinal and formulation-dependent fluctuations. Importantly, no sex-related differences in systemic exposure were observed for either route. The primary objective of the pharmacokinetic and tissue distribution studies was not to establish bioequivalence between formulations, but rather to characterize the disposition of intravenous IV-EVE relative to conventional Oral-EVE under standard preclinical dosing conditions. The Oral-EVE used throughout the study was PEG400-solubilized everolimus. Oral-EVE was administered by gavage using a formulation consistent with established preclinical studies and product labeling. Importantly, Oral-EVE administration requires fasting conditions because food is known to substantially alter absorption and systemic exposure. Clinical studies have demonstrated that food can reduce everolimus C_max_ by approximately 60% and significantly affect overall bioavailability. Consequently, oral dosing is routinely performed under controlled fasting conditions in both preclinical and clinical pharmacokinetic studies to minimize variability and ensure reproducible exposure. In contrast, intravenous administration bypasses gastrointestinal absorption and first-pass metabolism and therefore does not require fasting. This distinction reflects the inherent differences between oral and intravenous delivery rather than a study design bias.

Oral-EVE in rats produced a dose-related toxicity profile consistent with systemic mTOR inhibition. Across repeat-dose rat studies, the principal target organs were lymphoid tissues, lung, kidney, stomach, thyroid, and male reproductive organs. The FDA pharmacology review specifically states that oral RAD001/everolimus in rats identified the lymphoid organs, lungs, kidneys, stomach, thyroid, and reproductive organs, especially males, as target organs, with dose-dependent incidence and severity and effects mainly seen at 1.5 mg/kg/day [6]. In the 26-week oral gavage rat study, the high dose of 1.5 mg/kg/day, equivalent to approximately 9 mg/m^2^/day, produced clear toxicity. Findings included mortality in one male, decreased body weight and body weight gain, reduced food conversion efficiency, hemoconcentration with increased RBC, hemoglobin, and hematocrit, increased neutrophils, decreased lymphocytes and platelets, increased amylase and lipase, increased cholesterol and triglycerides, decreased albumin and A/G ratio, decreased iron and phosphorus, increased urine volume, and organ weight changes involving the kidneys, pituitary, thymus, testes, epididymides, seminal vesicles, prostate, uterus, liver, lungs, and spleen. Histopathology showed lymphoid atrophy/lymphocytolysis, renal hydronephrosis and tubular pigment, lung alveolar macrophage accumulation and perivascular lymphocytic infiltration, splenic hemosiderosis, stomach inflammation and mucosal hyperplasia/hypertrophy, thyroid follicular changes, and male reproductive toxicity with reduced sperm, germ-cell depletion, spermatid giant cells, and tubular vacuolation. For Oral-EVE, the FDA pharmacology/toxicology review also identified lung and heart toxicity. In the oral rat studies, histopathology showed diffuse alveolar macrophages in males at approximately 0.5 mg/kg/day and in females at 1.5 mg/kg/day, as well as chronic myocarditis at 1.5 mg/kg/day and myocardial fibrosis in one high-dose male. In the FDA pharmacology/toxicology review for Afinitor/RAD001, Oral-EVE rat toxicity included male reproductive organs as target organs. The rat findings were described as reduced sperm count, depletion of germ cells, partial depletion of one or more generations of germ cells, spermatid giant cells, and tubular vacuolation.

The gastrointestinal tract was a bona fide target in rats. GI toxicity in rats also emerged most clearly at 1.5 mg/kg/day, where the FDA review described stomach lesions consisting of acute inflammation in the glandular region with mucosal hyperplasia/hypertrophy. This also overlapped with decreased body weight, reduced food efficiency, hypoalbuminemia, and other systemic toxicity findings. The rat GI toxicity appears primarily gastric rather than intestinal. Intestinal erosion, mucosal atrophy, macrophage aggregation, and mucosal inflammation were more prominent in minipigs and monkeys, whereas the rat-specific lesion was stomach inflammation and mucosal hyperplasia/hypertrophy.

In summary, repeated administration of IV-EVE produced target organ toxicity involving the heart, thymus, lymphoid tissues, lungs, testes, and epididymides, while no gastric pathology was identified by clinical observations, gross pathology, or histopathology. This absence of gastric injury represents a major distinction from the toxicological profile of Oral-EVE and supports the hypothesis that intravenous delivery may mitigate gastrointestinal exposure-related toxicity while maintaining systemic pharmacological activity.

Although everolimus is an established mTOR inhibitor approved for multiple oncologic indications, including advanced renal cell carcinoma, HR-positive/HER2-negative metastatic breast cancer, pancreatic neuroendocrine tumors, and tuberous-sclerosis-associated neoplasms, its clinical use has been limited to oral administration because of its poor aqueous solubility and low oral bioavailability, which is typically reported to be approximately 15–20%. As a consequence, the development of an intravenous everolimus product has historically been constrained by formulation challenges associated with the highly hydrophobic nature of the molecule. IV-EVE addresses this limitation through the Deciparticle™ platform, which was developed through systematic screening of a mPEG-based polymer library for the ability to encapsulate everolimus and form stable nanoparticles suitable for intravenous administration. Among the polymers evaluated, mPEG-Chol was selected based on its favorable formulation characteristics, ability to generate uniform nanoparticles, and established biocompatibility profile. Importantly, the resulting formulation is not a simple solubilized drug product, solvent-based preparation, or conventional micellar system, but rather a reproducible nanoparticle formulation capable of supporting clinical-scale manufacturing and intravenous delivery [16].

The novelty of IV-EVE extends beyond formulation design and includes the successful translation of the platform into a clinic-ready product. Manufacturing was performed under cGMP conditions using a scalable process involving nanoparticle formation, sterile filtration, aseptic fill–finish, lyophilization, and reconstitution to a final intravenous formulation containing 4 mg/mL everolimus. The resulting clinical product demonstrated consistent particle size characteristics, sterility, refrigerated stability, acceptable in-use stability after reconstitution, and successful scale-up sufficient to support ongoing clinical development. These characteristics distinguish IV-EVE from previously described experimental everolimus formulations that have not advanced to clinical evaluation. Perhaps most importantly, the present toxicology findings suggest that intravenous administration may alter tissue exposure patterns relative to oral everolimus. Chronic oral everolimus administration in rats has been associated with gastric toxicity characterized by glandular stomach inflammation and mucosal hyperplasia/hypertrophy, consistent with substantial local gastric exposure. In contrast, no gastric pathology was identified in the present intravenous toxicology study despite systemic exposures sufficient to produce pharmacologically relevant effects in lymphoid tissues, the lungs, the heart, and reproductive organs.

Taken together, these results establish IV-EVE as a novel intravenous everolimus platform that combines a clinically scalable Deciparticle™ formulation with favorable manufacturability, potent antitumor activity, and a differentiated nonclinical safety profile. The ability to administer everolimus intravenously may provide an opportunity to overcome important limitations of oral therapy and warrants continued clinical investigation.

## 5. Conclusions

IV-EVE, a Deciparticle™ intravenous formulation of everolimus, demonstrated pharmacokinetic and disposition characteristics distinct from those of oral everolimus while maintaining comparable metabolic pathways. Intravenous administration produced substantially higher and more predictable systemic exposure, dose-proportional pharmacokinetics, and broad tissue distribution without the marked gastrointestinal accumulation observed following oral administration. Elimination occurred predominantly through metabolic biliary/fecal pathways, with minimal excretion of unchanged drug. These findings indicate that intravenous delivery can overcome key limitations associated with oral everolimus, including variable bioavailability and extensive gastrointestinal exposure. The favorable pharmacokinetic profile, together with the absence of gastric pathology in repeat-dose toxicology studies and successful translation into a clinically scalable formulation, supports the continued clinical evaluation of IV-EVE in patients with advanced malignancies.

## Figures and Tables

**Table 1:** EVE excretion.

Test Article	Dose (mg/kg)	Dose Route	Gender	Number of Samples	Total in Feces (%)	Total in Urine (%)
IV-EVE	3	IV	Male	2	0.693	0.015
			Female	2	0.795	0.013
Oral-EVE	3	Oral	Male	2	0.721	0.106
			Female	2	0.500	0.090

**Table 2:** EVE PK characteristics.

Species/Route	Test Article (Dose)/N(M/F)	C_max_ (ng/mL)	T_max_ (h)	AUC_0–t_ (h·ng/mL)	AUC_0–∞_ (h·ng/mL)	t½ (h)
Rat/IV	IV-EVE (3 mg/kg)/4 (2/2)	133.448/98.195	0.083/0.083	323.759/214.744	339.203/220.930	12.88/12.67
Rat/PO	Oral-EVE (3 mg/kg)/4 (2/2)	2.408/1.822	4.00/1.25	42.630/22.781	52.169/29.483	16.76/11.11

**Table 3:** IV-EVE dose-proportional PK parameters.

Dose	Sex	N	t½	T_max_	C_max_	AUC_(0–t)_	AUC_(0–∞)_	AUC_(0–∞)_/Dose	Vd	Cl	Vss
mg/kg			h	h	ng/mL	h·ng/mL	h·ng/mL	(h·ng/mL)/(mg/kg)	mL/kg	mL/h/kg	mL/kg
5	M	3	11.36 ± 1.09	0.083 ± 0.000	330 ± 7	723 ± 78	741 ± 80	148 ± 16	111,010 ± 9347	6806 ± 752	51,299 ± 1902
	F	3	12.98 ± 2.90	0.083 ± 0.000	300 ± 3	632 ± 178	663 ± 183	133 ± 37	142,649 ± 13,580	7923 ± 2108	59,191 ± 9568
10	M	3	19.97 ± 3.12	0.083 ± 0.000	737 ± 33	1665 ± 203	1729 ± 232	173 ± 23	167,714 ± 27,855	5851 ± 762	44,911 ± 5623
	F	3	12.95 ± 5.65	0.083 ± 0.000	632 ± 83	1412 ± 176	1436 ± 187	144 ± 19	127,274 ± 39,295	7040 ± 867	39,525 ± 2524
15	M	3	8.74 ± 1.56	0.083 ± 0.000	900 ± 88	2377 ± 385	2421 ± 398	161 ± 27	78,407 ± 8815	6321 ± 1143	37,221 ± 5645
	F	3	9.07 ± 7.02	0.083 ± 0.000	1103 ± 220	2457 ± 244	2474 ± 232	165 ± 15	83,093 ± 71,074	6098 ± 570	28,433 ± 7614

**Table 4:** IV-EVE-related histopathological examination.

Group		Dosing Period (Day 30)				Recovery Period (Day 58)			
		1	2	3	4	1	2	3	4
Dosage (mg/kg)		0	5	10	15	0	5	10	15
No. Examined Animals (M/F)		10/10	10/10	10/10	10/10	5/5	5/5	5/5	5/5
Tissues and Lesions	Grade	Number of Occurrence (M/F)							
Thymus	No. Examined	10/10	10/10	10/10	10/10	5/5	5/5	5/5	5/5
Cellularity, decreased, lymphocytes, cortex	Minimal	0/0	6/4	7/4	7/6	0/0	0/0	0/0	0/0
	Mild	0/0	4/0	1/0	1/0	0/0	0/0	0/0	0/0
	Total	0/0	10/4	8/4	8/6	0/0	0/0	0/0	0/0
Lymph node, mandibular	No. Examined	10/10	10/10	10/10	10/10	5/5	5/5	5/5	5/5
Decreased size and number, germinal centers	Minimal	0/0	6/5	3/3	4/3	0/0	0/0	0/0	0/0
	Mild	0/0	1/0	2/1	2/3	0/0	0/0	0/0	0/0
	Total	0/0	7/5	5/4	6/6	0/0	0/0	0/0	0/0
Lymph node, mesenteric	No. Examined	10/10	10/10	10/10	10/10	5/5	5/5	5/5	5/5
Decreased size and number, germinal centers	Minimal	0/0	4/4	3/4	5/4	0/0	0/0	0/0	0/0
	Mild	0/0	2/1	2/1	2/2	0/0	0/0	0/0	0/0
	Total	0/0	6/5	5/5	7/6	0/0	0/0	0/0	0/0
Tingible body macrophage, cortex	Minimal	0/0	1/1	0/0	0/0	0/0	0/0	0/0	0/0
	Mild	0/0	1/0	0/0	2/0	0/0	0/0	0/0	0/0
	Total	0/0	2/1	0/0	2/0	0/0	0/0	0/0	0/0
Lung (with main stern bronchi)	No. Examined	10/10	10/10	10/10	10/10	5/5	5/5	5/5	5/5
Aggregation, foamy macrophage, alveolar	Minimal	0/0	6/5	6/8	1/7	0/0	1/2	1/2	1/0
	Mild	0/0	1/0	0/0	5/1	0/0	0/1	1/1	2/1
	Total	0/0	7/5	6/8	6/8	0/0	1/3	2/3	3/1
Heart	No. Examined	10/10	10/10	10/10	10/10	5/5	5/5	5/5	5/5
Necrosis/inflammatory cell infiltrate, myocardium	Minimal	0/0	8/1	4/3	4/2	0/0	0/0	0/0	0/0
	Total	0/0	8/1	4/3	4/2	0/0	0/0	0/0	0/0
Mononuclear cell infiltrate/fibrosis, myocardium	Minimal	0/0	0/0	0/0	0/0	0/0	2/1	1/0	0/0
	Total	0/0	0/0	0/0	0/0	0/0	2/1	1/0	0/0
Testes	No. Examined	10/-	10/-	10/-	10/-	5/-	5/-	5/-	5/-
Degeneration, germ cell, seminiferous tubules	Minimal	0/-	5/-	4/-	4/-	0/-	0/-	0/-	0/-
	Mild	0/-	5/-	5/-	6/-	0/-	1/-	0/-	1/-
	Moderate	0/-	0/-	1/-	0/-	0/-	2/-	0/-	0/-
	Total	0/-	10/-	10/-	10/-	0/-	3/-	0/-	1/-
Degeneration/atrophy, seminiferous tubules	Mild	0/-	0/-	0/-	0/-	0/-	0/-	2/-	0/-
	Moderate	0/-	0/-	0/-	0/-	0/-	2/-	3/-	4/-
	Total	0/-	0/-	0/-	0/-	0/-	2/-	5/-	4/-
Epididymides	No. Examined	10/-	10/-	10/-	10/-	5/-	5/-	5/-	5/-
Cell debris, luminal	Minimal	0/-	5/-	8/-	8/-	0/-	1/-	1/-	2/-
	Mild	0/-	1/-	1/-	0/-	0/-	4/-	4/-	3/-
	Total	0/-	6/-	9/-	8/-	0/-	5/-	5/-	5/-
Reduced sperm, luminal	Minimal	0/-	8/-	9/-	8/-	0/-	0/-	0/-	1/-
	Mild	0/-	0/-	1/-	1/-	0/-	3/-	3/-	0/-
	Moderate	0/-	0/-	0/-	0/-	0/-	2/-	2/-	4/-
	Total	0/-	8/-	10/-	9/-	0/-	5/-	5/-	5/-

## Data Availability

The data presented in this study are available upon reasonable request from the corresponding author due to proprietary confidentiality considerations.

## Abbreviations

The following abbreviations are used in this manuscript:

EVE	Everolimus
API	Active Pharmaceutical Ingredient
IV	Intravenous
PK	Pharmacokinetics
